# Supplementary material for: Psychological, sociocultural and economic coping strategies of mothers or female caregivers of children with a disability in Belu district, Indonesia
Source: PLoS One. 2021 May 6;16(5):e0251274. doi: 10.1371/journal.pone.0251274 (PMC8101963; doi:10.1371/journal.pone.0251274)
Supplement: Attachment — Submitted filename: Response to Reviewers.docx [file pone.0251274.s002.docx]

**Response to Reviewers**

Thank you so much for considering our manuscript to be published in PLOS ONE. Herewith we submit the revised version of our paper entitled ‘**Psychological, sociocultural and economic coping strategies of mothers or female caregivers of children with a disability in Belu District, Indonesia’**

On behalf of all authors,

Sincerely,

Nelsensius Klau Fauk

1. Please ensure that your manuscript meets PLOS ONE's style requirements, including those for file naming.

**Response:**

The manuscript has been adjusted to the journal guidelines.

2. Methodology: Informative. However, authors should indicate how many participants whose children reside in the rehabilitation centres and how many parents who stay with their children?

**Response:**

Of the 22 participants in this study, 15 participants stay with their children with a disability and 7 participants’ children reside in the rehabilitation centres.

3. Why was snowball used because the principal and the leaders of the 2 rehabilitation centres know the parents or caregivers of these children or have records of their contact details? Meaning they should have contacted them to distribute the information sheet rather than the researchers asking the initial participants.

**Response:**

To avoid the misuse of power of the school Principal and the leaders of the rehabilitation centres which could make the mothers or caregivers feel obliged to participate, we only asked for their help to post the study information sheet on their information board which could be reached or read by the potential participants:

- Participants recruitment processes employed a combination of purposive and snowball sampling techniques, starting with enlisting the help of the Principal of the state special school and the leaders of the two rehabilitation centres to distribute the study information sheet containing the contact detail of the field researcher (GAA) to potential participants by posting it on the information board at the school and rehabilitation centres [8]. This was followed by the snowball sampling technique where the initial participants who called to confirm their participation and were interviewed, were also asked to distribute the study information sheet to their friends, colleagues or other potential participants who might be willing to participate in this study.

4. References: Many references (>60%) were published before 2016

**Response**

The references have been updated.
